# Exploring the Potential of Selenium-Containing Amine (Se-AMA) to Enhance Photosynthesis and Leaf Water Content: New Avenues for Carbonic Anhydrase Modulation in *Arabidopsis thaliana*

**DOI:** 10.3390/plants14020258

**Published:** 2025-01-17

**Authors:** Sara Beltrami, Francesca Alderotti, Antonella Capperucci, Damiano Tanini, Cecilia Brunetti, Francesco Ferrini, Ermes Lo Piccolo, Antonella Gori

**Affiliations:** 1 Department of Agriculture, Food, Environment and Forestry (DAGRI), University of Florence, Viale delle idee 30, 50019 Sesto Fiorentino, Florence, Italy; sara.beltrami@unifi.it (S.B.); francesco.ferrini@unifi.it (F.F.); ermes.lopiccolo@unifi.it (E.L.P.); antonella.gori@unifi.it (A.G.); 2 Institute for Sustainable Plant Protection, National Research Council of Italy (CNR), Via Madonna del Piano 10, 50019 Sesto Fiorentino, Florence, Italy; francesca.alderotti@cnr.it; 3 Department of Chemistry ‘Ugo Schiff’, University of Florence, Via Della Lastruccia 3–13, 50019 Sesto Fiorentino, Florence, Italy; antonella.capperucci@unifi.it (A.C.); damiano.tanini@unifi.it (D.T.)

**Keywords:** apparent carboxylation efficiency, leaf gas exchange, net photosynthesis, synthetic activators, stomatal conductance

## Abstract

Global changes and growing demands have led to the development of new molecular approaches to improve crop physiological performances. Carbonic anhydrase (CA) enzymes, ubiquitous across various life kingdoms, stand out for their critical roles in plant photosynthesis and water relations. We hypothesize that the modulators of human CAs could affect plant physiology. Our research demonstrated that foliar treatments with a synthetic selenium-containing CA activator (Se-AMA) influenced the physiological performances of *Arabidopsis thaliana*. Se-AMA increased net photosynthesis (A + 31.7%) and stomatal conductance (g_s_ + 48.2%) at 100 µM, with the most notable effects after 10 days of treatment. Se-AMA at 300 µM proved to be even more effective, boosting A and g_s_ by 19.9% and 55.3%, respectively, already after 3 days of application. Morning treatment with Se-AMA at 300 µM enhanced photosynthetic performances throughout the day, suggesting that the positive effect of Se-AMA lasted for several hours. Additionally, Se-AMA increased water content in plants by 17.1%, suggesting that Se-AMA treatment may have improved plant water absorption and resource management. This effect might be linked to Se-AMA’s role in modulating specific CA isoforms working with aquaporins. Although preliminary, these findings suggest that Se-AMA could enhance plant physiological performances under the conditions of non-limiting water availability.

## 1. Introduction

The impacts of global change, such as increased temperatures and reduction in arable lands and water availability, are severe constraints threatening plant productivity [1]. This, together with the rise in human population and bioenergy demands, addresses the need for continuously improving crop physiological performances. The current efforts to fulfill this objective are, nowadays, focusing on enhancing plant photosynthesis. Among the different approaches exploited by researchers to optimize photosynthesis, the bioengineering of crops led to very encouraging results, demonstrating that the manipulation of genes encoding proteins crucial for the Calvin–Benson cycle, such as RuBisCO, can potentially enhance crop yields [2]. Besides the advancements in genetic engineering, new opportunities are now available. One promising approach relies on implementing carbon dioxide (CO_2_) assimilation by boosting the activity of carbonic anhydrases (CAs), metalloenzymes that catalyze the reversible conversion of CO_2_ to HCO_3_^−^ and facilitate the movements of CO_2_ and HCO_3_^−^ across membranes [3].

Primarily discovered in blood cells, CAs are currently recognized as ubiquitous across various kingdoms of life, accounting for up to 2% of the total soluble protein in plant leaves [3,4]. In C3 species, three evolutionarily different isoforms of CAs are present: alpha (α), beta (β), and gamma (γ) [5]. Most of the total CA activity is in the chloroplast matrix [6], where α and β isoforms (found in both granal and stromal thylakoid membranes) are thought to improve inorganic carbon acquisition and fixation by facilitating the effective passage of inorganic carbon through the liquid phase and maintain the concentration of CO_2_ at the site of RuBisCO [7,8,9,10]. Along with chloroplast CAs, those located in the cell wall and plasma membrane are also considered important for the photosynthetic process because they may interact with aquaporins (AQPs) at membrane–liquid interfaces, further facilitating CO_2_ transfer as HCO_3_^−^ [11,12,13]. Thus, the involvement of various CAs in cooperatively supplying inorganic carbon to carboxylation sites and plant water status appears well supported [9,13,14].

However, despite its potential applicative importance, the molecular modulation of carbonic anhydrases in plants has been poorly investigated so far. On the other hand, owing to their key role in a broad array of biological processes ranging from bone reabsorption and vascular calcification to tumorigenesis, human CAs have been widely studied [15]. Studies on animal CA isoforms highlighted that a number of organic compounds, characterized by well-defined structural motives, could behave as enzyme inhibitors or activators [16,17,18,19]. In particular, CA activators have been demonstrated to increase the CA activity by facilitating the proton transfer reaction between the metal ion center and the external medium, generating the active form of the enzyme. In this context, human CAs have become important pharmacological targets, and a number of highly efficient and selective modulators have been developed and are available for therapeutic applications [20,21].

Taking inspiration from the research on animal CAs, we propose to use a CA modulator as a new potential tool to improve plant photosynthetic performances, likely increasing their biomass. Based on an accurate literature survey, amines and amino acids appeared the most interesting classes of small molecules to be employed as CA activators [18,22]. Indeed, the activation profile as well as the structure–activity relationships of differently substituted amines and amino acids, including heteroatom-substituted systems, have been extensively investigated during the last decade [23]. Furthermore, the low (or null) toxicity, together with their relatively easy and cheap synthesis, made this class of small molecules even more attractive for our application purposes [18,21,24].

Hence, we synthesized a CA activator, the selenium-containing amine (Se-AMA), and evaluated its effects on the model plant *Arabidopsis thaliana*, with a particular focus on leaf physiological performances and biomass accumulation. The primary objectives of the study are to evaluate the effect of the molecule on plant physiology under standard growing conditions, with an emphasis on determining the optimal doses and application methods to achieve the best possible results. Thus, three different experiments were performed. Firstly, the active concentration range was selected, testing various concentrations and their timing of action. Additionally, the impact of high doses was observed to see whether the effects could be triggered more rapidly. Finally, the effects of a single Se-AMA treatment throughout the day were examined to assess the endurance of the molecule’s activity.

## 2. Results

### 2.1. Plant Physiological Responses to Different Doses of Se-AMA (Experiment 1)

**Figure 1:**
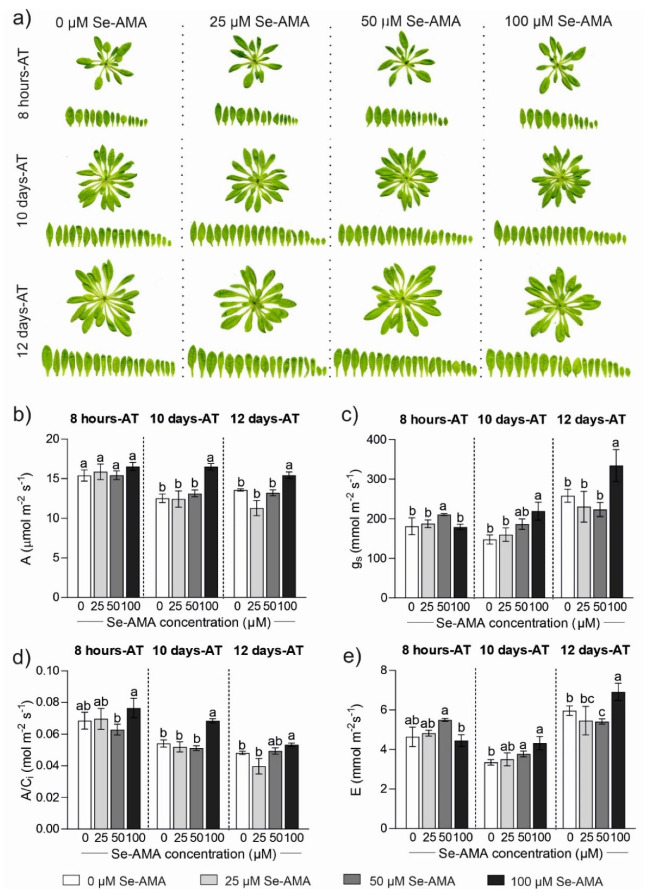
Impact of different doses of Se-AMA on the physiological performances of *Arabidopsis thaliana* (experiment 1). Three separate groups of plants were treated with foliar sprays containing varying concentrations of the tested molecule (25, 50, or 100 µM of Se-AMA dissolved in an aqueous solution). An additional group of plants was sprayed with a mock solution (without Se-AMA) to serve as controls (0 µM of Se-AMA). During the experiment, the plants were monitored for phytotoxic symptoms (**a**) and subjected to a gas exchange analysis, which included the measurements of net photosynthesis (A) (**b**), stomatal conductance (g_s_) (**c**), apparent carboxylation efficiency (A/C_i_) (**d**), and transpiration rate (E) (**e**). Three time points were assessed: 8 h after treatment (AT), 10 days-AT, and 12 days-AT. Data are presented as means ± SD from four biological replicates, with different letters indicating significant differences between the Se-AMA treatments at each time point (two-way ANOVA with repeated measurements, followed by a post hoc Tukey test, *p* < 0.05, *n* = 4). A comprehensive summary of the statistical analysis is provided in Table S1, including details on the effect of time on the photosynthetic performances for each treatment.

*Arabidopsis thaliana* photosynthetic performances were influenced by Se-AMA without negatively affecting plant health status, as no chlorotic or necrotic areas were detected on the treated leaves throughout the first experiment (Figure 1a). Our results showed that a single application of Se-AMA was incapable of enhancing plant photosynthetic performance, at least within the first 8 h of treatment (Figure 1b–e and Figure S1). In this time span, only the 50 µM Se-AMA-treated plants displayed a higher stomatal conductance (g_s_, mmol m^−2^ s^−1^) compared to the control ones, but no differences were observed in all the remaining treatments (0, 25, and 100 µM Se-AMA) (Figure 1c). Interestingly, we found that a higher dose of Se-AMA (100 µM) was effective in improving plant photosynthetic performances starting from 10 days after treatment (10 days-AT) to the end of the experiment (12 days-AT), whereas lower doses of the molecule (25 and 50 µM Se-AMA) were unable to provide any effect. At 10 days-AT, the 100 µM Se-AMA-treated plants were more photosynthetically active than the control plants, displaying a significant increase in the net photosynthesis (A, µmol m^−2^ s^−1^) and g_s_ of 31.7% and 48.2%, respectively (Figure 1b,c). Though less evident, the positive effect of Se-AMA on photosynthesis was still present after 12 days of application, with the plants supplied with 100 µM Se-AMA showing an increase in both g_s_ and A of 29.6% and 13.8% than the controls, respectively (Figure 1b,c). Compared to the control treated plants, all the Se-AMA treatments (25, 50, and 100 µM) did not significantly change the intracellular CO_2_ (C_i_, µmol m^−2^ s^−1^) along the first experiment, except for the 50 µM treatment showing a slightly higher C_i_ at 10 days-AT (Figure S1a). By analyzing the photosynthesis response in relation to the amount of CO_2_ accumulated at the sub-stomatal level (A/C_i_, mol m^−2^ s^−1^), we found that the apparent carboxylation efficiency was increased in the plants treated with 100 µM Se-AMA, while no differences were observed between the other treatments and the control (Figure 1d). Specifically, a slight increase in A/C_i_ was observed as early as 8 h after the first application of the molecule at 100 µM, although the most significant results were obtained after 10 days of treatment when A/C_i_ in plants treated with 100 µM Se-AMA was increased by 26.4% compared to the other treatments (Figure 1d). In line with the smaller increase in A induced by Se-AMA following prolonged treatment, the enhancement in A/C_i_ was less pronounced at 12 days-AT, stabilizing around 10% above the control condition (Figure 1a,d).

Regarding transpiration, a significant increase was observed starting from 10 days-AT, with the plants treated with 50 and 100 µM Se-AMA showing a 12.9% and 28.9% higher transpiration rate (E), respectively, compared to the control plants (Figure 1e). At 12 days-AT, E decreased in the 50 µM treatment, but remained elevated in the 100 µM treatment, showing a 15.8% increase compared to the control. Besides Se-AMA clearly affected transpiration mainly through stomatal regulation, less evident effects were observed in terms of water use efficiency (WUE_i_) (Figure S1b). The 50 µM Se-AMA treatment was the only one to alter this parameter, significantly decreasing WUE_i_ at 10 days-AT while promoting its increase at 12 days-AT. Overall, our results indicated that the foliar application of Se-AMA can improve the photosynthetic performance of *A. thaliana*, preferentially affecting A and g_s_, with high application rates of Se-AMA (i.e., 100 µM) promoting the most remarkable effects.

**Figure 2:**
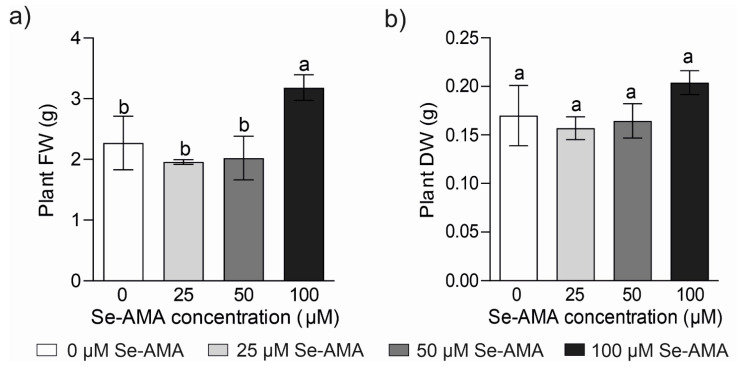
Effect of different doses of Se-AMA application on *Arabidopsis thaliana* water content and dry matter (Experiment 1). After 10 days of treatments, parameters relative to plant fresh weight (FW) and plant dry weight (DW) on the plants treated either with a control solution (0 µM Se-AMA) or with different concentrations of Se-AMA (25, 50, or 100 µM) were assessed: FW (**a**) and DW (**b**). Data are presented as means ± SD from four biological replicates, with different letters indicating significant differences between the Se-AMA treatments (one-way ANOVA, followed by a post hoc Tukey test, *p* < 0.05, *n* = 4).

In terms of plant growth, high doses of Se-AMA (100 µM) remarkably influenced plant moisture after 12 days of application, increasing plant fresh weight and plant water content by 20.0% and 41.7%, respectively (Figure 2a and Figure S1a). Since the Se-AMA treatment did not cause significant changes in plant dry weight, the dry matter displayed by the plants treated with 100 µM Se-AMA was lower than that of the control group (Figure 2b and Figure S2b). Finally, comparable values of the plant area and plant mass per area were recorded in both the treated and control plants at 12 days-AT (Figure S2c,d).

### 2.2. Effect of High Doses of Se-AMA on Plant Physiological Performances (Experiment 2)

**Figure 3:**
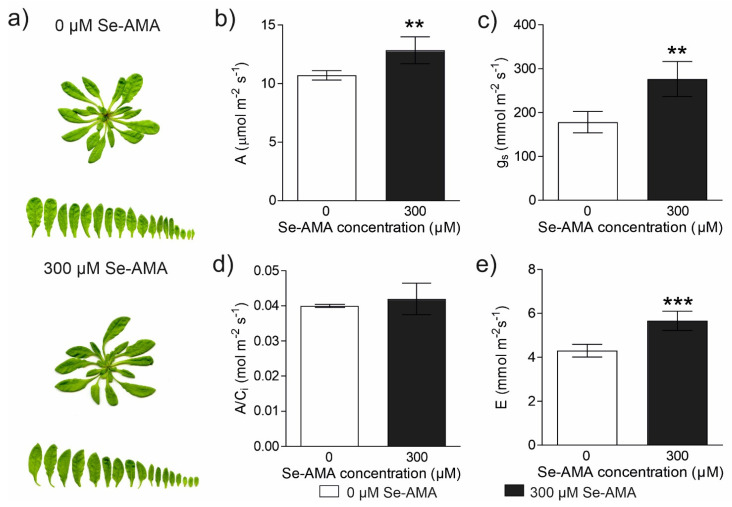
Impact of high doses of Se-AMA on the physiological performances of *Arabidopsis thaliana* (experiment 2). Treatment was performed by spraying the plants with an aqueous solution containing 300 µM Se-AMA for three consecutive days. A mock solution (without Se-AMA) was applied to an additional group of plants serving as controls (0 µM of Se-AMA). At three days after treatment (AT), the plants were monitored for phytotoxic symptoms (**a**) and subjected to a gas exchange analysis: net photosynthesis (A) (**b**), stomatal conductance (g_s_) (**c**), apparent carboxylation efficiency (A/C_i_) (**d**), and transpiration rate (E) (**e**). Data are presented as means ± SD, with stars indicating significant differences between the control and Se-AMA treatments (unpaired *t*-test, ** *p* < 0.01, *** *p* < 0.001, *n* = 5).

The effects of 300 µM Se-AMA were very similar to that induced by the 100 µM solution, with Se-AMA improving plant net photosynthesis without negatively altering the plant health status (Figure 3 and Figure S3). Actually, the high concentration of Se-AMA applied over three consecutive days did not induce any phytotoxicity symptoms on plant leaves, as similar levels of chlorophyll and polyphenols were observed in both the treated and control plants (Figure S3a–c). Also, the nitrogen balance index (NBI) remained unaffected by the Se-AMA treatment, strongly highlighting the non-toxic behavior of this selenium-containing amine in plants (Figure S3d).

When compared to the controls, A and g_s_ increased in the plants supplied with 300 µM Se-AMA by 19.9% and 55.3%, respectively (Figure 3b,c). Interestingly, a significant increase in C_i_ was observed in the treated plants at 3 days-AT, while no changes were detected in the A/C_i_ ratio or in the F_v_/F_m_ ratio (Figure 3d, Figures S4a, and S5). Additionally, the 300 µM treatment influenced E and WUE_i_, resulting in a 31.6% increase in E and a 22.8% decrease in WUE_i_ (Figure 3e and Figure S4b).

**Figure 4:**
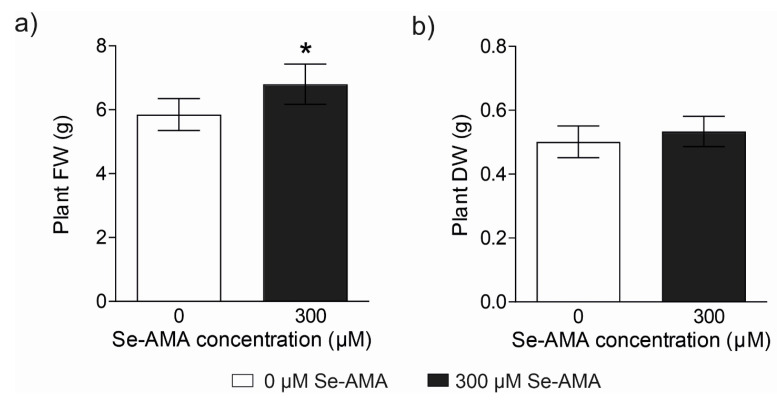
Impact of high doses of Se-AMA on *Arabidopsis thaliana* growth. At 3 days after treatment (AT), growth-related parameters of both controls (0 µM Se-AMA) and plants supplied with 300 µM Se-AMA were assessed: plant fresh weight (FW) (**a**) and plant dry weight (DW) (**b**). Data are presented as means ± SD, with stars indicating significant differences between control and Se-AMA treatments (unpaired *t*-test, * *p* < 0.05, *n* = 5).

Regarding plant growth, the effect of Se-AMA at higher doses resulted in increased plant fresh weight, higher water content, and reduced dry matter in the treated plants compared to the controls (Figure 4a and Figure S6a,b). In contrast, the plant DW, plant area, and plant mass area remained unchanged (Figure 4b and Figure S6c,d).

### 2.3. Daily Effect of Se-AMA on Plant Physiological Performances (Experiment 3)

**Figure 5:**
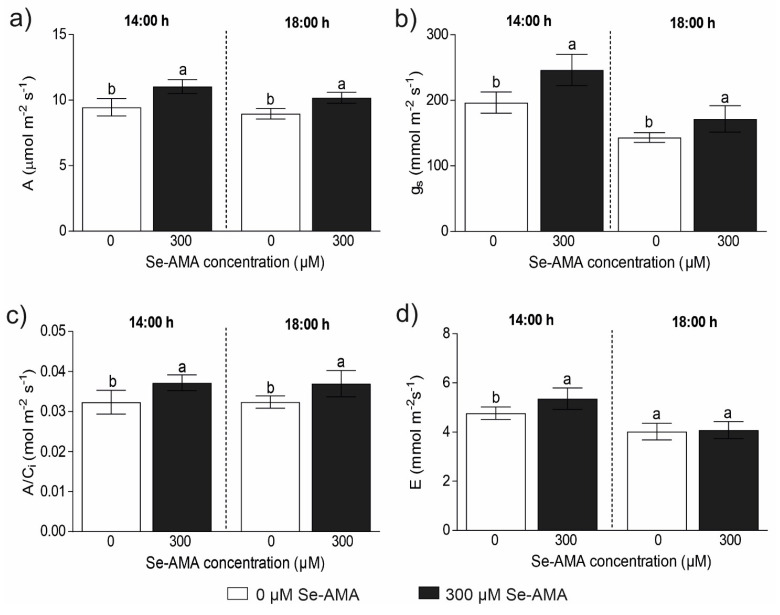
Daily effect of Se-AMA on the physiological performances of *Arabidopsis thaliana* (experiment 3). The impact of a single treatment with 300 µM Se-AMA on daily photosynthetic performances was assessed by performing a gas exchange analysis at 14:00 h (h) and 18:00 h: net photosynthesis (A) (**a**), stomatal conductance (g_s_) (**b**), apparent carboxylation efficiency (A/C_i_) (**c**), and transpiration rate (E) (**d**) of the plants either mock-treated (0 µM Se-AMA) or supplied with Se-AMA (300 µM Se-AMA). Data are presented as means ± SD, with different letters indicating significant differences between the Se-AMA treatments at each time point (two-way ANOVA with repeated measurements, followed by a post hoc Tukey test, *p* < 0.05, *n* = 5). A comprehensive summary of the statistical analysis is provided in Table S2, including details on the effect of time on photosynthetic performances for each treatment.

A single treatment with Se-AMA at 300 µM performed in the morning enhanced plant photosynthetic performances throughout the day (Figure 5), confirming that the positive effect of Se-AMA on photosynthesis can last several hours from its application. In particular, A of the treated plants increased by 16.7% in the afternoon and was still 13.4% higher than in the control plants at sunset (Figure 5a). A quite similar effect was observed on g_s_, which was significantly increased by Se-AMA at both times of the day (Figure 5b). Compared to the control, the plants treated with 300 µM Se-AMA displayed 1.25-fold more g_s_ at 14:00 h and significantly higher g_s_ also at 18.00 h (Figure 5b). A single application of Se-AMA at 300 µM also enhanced A/C_i_, leading to an average daily increase in the A/C_i_ ratio of approximately 14–15% (Figure 5c). Regarding transpiration, E only increased at 14:00 h, likely reflecting the maximal g_s_ achieved in the Se-AMA-treated plants at this time of day (Figure 5b,d). No significant differences were observed in C_i_ or WUE_i_ throughout the day between the control and treated plants (Figure S7), which also displayed similar levels of F_v_/F_m_ (Figure S8).

## 3. Discussion

Changes in climate represent one of the biggest threats the world is now facing. The alterations in climatic conditions, such as increasing temperature and changes in precipitation patterns, have severely dampened crop yield and biomass production, causing important economic losses in both agricultural and forestry sectors [25,26]. So far, great effort has been successfully placed into developing mitigation strategies aimed at increasing plant physiological performances in the field, primarily focusing on the genetic optimization of photosynthetic processes [27,28]. Besides genetic engineering, novel alternative strategies exist to improve photosynthesis [29]. Among them, the optimization of carbon dioxide (CO_2_) assimilation through the modulation of carbonic anhydrases (CAs) appears very promising. Activation studies conducted on animal CAs highlighted that several organic compounds, known as CA activators, can fasten the CA-catalyzed hydration of CO_2_ by directly binding to the active site of the enzyme [16]. Since carbon anhydrases present in different organisms appeared to share a similar catalytic mechanism [3], we hypothesized that the activity of carbon anhydrases in plants could be improved by the application of CA activator analogs. Therefore, supported by the literature, we synthesized a CA activator (CAA) and evaluated its potential to influence plant physiological performances. Literature-reported data on human α-CAs and bacterial β-CAs provided important indications on the structural motives required to develop an effective CA activator. Amongst a range of organic compounds that can be employed, substitute amines were selected [18,22], due both to their ability to improve the catalytic efficiency of different isoforms of CAs and to their low toxicity [16,23,29]. The presence of the free amino functionality and of an aromatic ring—with a suitable chain spacer between these groups—seems to be crucial for the biological activity of such derivatives [16,23]. In this context, on the basis of our previous reports on sulfur- and selenium-containing amines [19,23], we envisaged the aminoselenide Se-AMA as the target compound of this study.

**Scheme 1:**
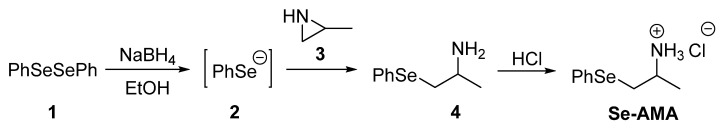
Synthesis of Se-AMA.

The synthetic strategy to access the target aminoselenide SeAMA relies on the nucleophilic ring opening reaction (NROR) of a N-*H* unactivated aziridine with a suitable selenium-centered nucleophilic partner. NRORs of strained heterocycles such as epoxides, thiiranes, and aziridines have been demonstrated to be versatile and effective routes for the synthesis of variously functionalized sulfur-, selenium-, and tellurium-containing small molecules [30]. Herein, aminoselenide **4** was easily prepared via the reaction of aziridine **3** with selenolate **2**, readily generated in situ upon the reduction of the parent diselenide **1** with sodium borohydride. In order to improve its solubility in water, aminoselenide **4** was converted into its hydrochloride Se-AMA upon treatment with an equimolar amount of hydrochloric acid (Scheme 1).

Notably, the tested molecule displayed good solubility in water and excellent chemical stability, highlighting the possibility of studying the effects of its application on plants over 30 days.

### 3.1. Effect of Se-AMA on Plant Physiological Performances

We found that the foliar spray of Se-AMA positively affected the physiological performances of *Arabidopsis thaliana* without causing any phytotoxicity to the plant (Figure 1). This was evidenced by the similar levels of chlorophyll and maximum quantum efficiency of Photosystem II (F_v_/F_m_) observed in both the treated and control leaves, as well as by the NBI, which remained unchanged by the Se-AMA treatment (Figures S3 and S5). Moreover, the leaves treated with the molecule showed no evidence of oxidative stress, as indicated by comparable levels of flavonoids and anthocyanins in both the treated and control plants (Figure S3). The most remarkable effects of Se-AMA were observed on A and g_s_, which were particularly enhanced by the 100 µM Se-AMA treatment after 10 days of administration (Figure 1). Two more days of the same treatment induced an even higher improvement in photosynthetic performances, indicating that Se-AMA can influence plant physiological status depending on the extent of its application. Furthermore, no significant effects on plant eco-physiological responses were induced by lower doses of Se-AMA (25 and 50 µM), indicating 100 µM as the minimum effective concentration for the tested molecule. Interestingly, we found that a 3-fold higher concentration of Se-AMA (300 µM) required a shorter period of application (three days) to improve the photosynthetic performances of the treated plants (Figure 3). Additionally, the plants subjected to a single treatment with 300 µM Se-AMA in the morning displayed enhanced photosynthesis and better apparent carboxylation efficiency as well as high stomatal conductance throughout the entire day (Figure 5), indicating that the effect of Se-AMA on plant photosynthetic performances can last for several hours. These results suggest that Se-AMA can positively influence plant physiological performances, affecting single photosynthetic parameters differently depending on the dose and time of its application. Nevertheless, the increased photosynthetic capacity resulting from the Se-AMA application did not significantly impact dry biomass production in *A. thaliana* plants, as evidenced by the not significant differences in dry weight observed between the treated and control plants (Figure 2 and Figure 4). It is important that in our study, the plants were treated with micromolar concentrations of Se-AMA, and biomass was evaluated only after 3 or 12 days of treatment. This may indicate that higher doses and/or extended application durations may be required to detect any measurable effects of Se-AMA on plant dry biomass.

Changes in stomatal conductance are among the most critical responses of leaves to fluctuations in environmental conditions [12]. The regulation of stomatal aperture facilitates CO_2_ diffusion into the intercellular air space (C_i_), thereby enhancing photosynthetic efficiency [31]. In our study, although low concentrations of the molecule led to an increase in g_s_, they did not cause a notable rise in C_i_. This observation suggests that the enhanced photosynthetic activity in the treated plants may be attributed to an increased A/C_i_ ratio. This hypothesis was validated in the 100 µM treatment, where the increase in A was accompanied by a substantial elevation in the A/C_i_ ratio (Figure 1). Consistent results were observed with a single application of Se-AMA at 300 µM (Figure 5), supporting that beyond stomatal conductance, additional non-stomatal mechanisms may contribute to the observed improvements in photosynthesis in the Se-AMA-treated plants. Among these factors, mesophyll conductance (g_m_)—defined as the diffusion of gases from sub-stomatal cavities to the chloroplast stroma—emerged as a particularly important candidate, as it has already been shown to work alongside g_s_ in regulating photosynthesis [31,32]. While the metabolic processes influencing g_m_ remain unclear, a growing body of experimental evidence suggests that carbonic anhydrases may serve as the potential regulators of resistance to CO_2_ diffusion within the mesophyll [11,33]. When Se-AMA was applied at 300 µM for several days, the increase in A and g_s_ induced in the treated plants was not associated with a significant increase in the A/C_i_ ratio, indicating that photosynthesis may be more significantly influenced by stomatal factors. However, it cannot be excluded that Se-AMA may still exert an effect on g_m_. Thus, we hypothesize that Se-AMA primarily may influence g_m,_ leading to an increased A/C_i_ ratio in the short period (Figure 5), while over time, the effect of Se-AMA on g_s_ may prevail over its effect on g_m_, leading to an increase in A. However, further investigations are needed to confirm this hypothesis and clarify whether and which specific carbon anhydrases are involved in this process. Activation studies of isolated CA isoforms with Se-AMA, along with experiments using *A. thaliana* mutants deficient in CA activity, will aid us in addressing this issue.

### 3.2. Effect of Se-AMA on Plant Water Status

While increased stomatal opening is beneficial for photosynthesis, it also leads to higher transpiration rates and, consequently, greater water loss, with a risk of dehydration [32,34]. Despite exhibiting high levels of transpiration (Figure 1 and Figure 3), the Se-AMA-treated plants were more hydrated than the control ones, showing greater water accumulation and fresh weight gain (Figure 2, Figure 4, Figures S2, and S6). We, thus, propose that Se-AMA application may enhance water absorption, likely increasing leaf hydraulic conductivity via transpiration under non-limited water availability [35,36]. This might be achieved through an association between specific CAs and aquaporin isoforms, important protein water channels primarily involved in the regulation of water flow within the plant [37,38]. A similar coordinated regulation has already been reported for βCA4 and PIP2.1 in *A. thaliana*, as well as for ZmCA4 and ZmPIP2;6 in maize [13,37]. Additionally, the expression of genes encoding CAs and aquaporins has been found to overlap in olive trees under water stress [11], reinforcing the concept of a functional interplay between CAs and aquaporins. Nonetheless, additional experiments are necessary to understand better the extent to which CAs, aquaporins, and their interaction may contribute to the observed effect in plants treated with Se-AMA. Although the molecular mechanism remains elusive, the effects of Se-AMA on plant transpiration could have important practical implications in the context of global warming. Recent evidence indicates that plants respond to high temperatures by increasing their transpiration rates, likely as a ‘heat avoidance’ strategy that depends on latent evaporative cooling [39]. Therefore, treating plants with Se-AMA could be a potential strategy to increase plant tolerance to high temperatures in conditions of non-limiting water availability.

## 4. Conclusions

Our study revealed the potential of a selenium-containing amine (Se-AMA) to enhance the physiological performances of *A. thaliana*. Overall, our results showed that the foliar application of the tested molecule at two different concentrations (100 and 300 μM) induced a dose-dependent increase in net photosynthesis (A) and stomatal conductance (g_s_) without negatively affecting plant health status. Interestingly, a single application of high doses of Se-AMA in the morning promoted the improvement of photosynthetic performances throughout the day, suggesting a rapid and long-lasting activity of the tested molecule. Furthermore, the Se-AMA treatments increased the plant water content without significantly altering dry biomass accumulation. Although the exact mechanism of action of the molecule remains to be fully elucidated, we speculate that the positive effects of Se-AMA on *Arabidopsis thaliana* might result from the activation of specific CA isoforms which, probably in association with some particular protein channels like aquaporins, can modulate the transfer of both HCO_3_^−^ and water across cell membranes. Our findings are preliminary and need to be tested with other experiments to confirm the potential of Se-AMA on the activity of specific CA isoforms and aquaporins. In addition, future experiments could be performed on crop species in conditions of limited water availability, paving the way for new research aimed at enhancing crop physiology in response to climate change.

## 5. Materials and Methods

### 5.1. Plant Material and Growth Conditions

*Arabidopsis thaliana*, ecotype Columbia 0 (Col-0), plants were cultivated in the growth chamber of DAGRI-UNIFI (Italy, Sesto Fiorentino, 43°50′ N, 11°12′ E) under controlled conditions: 120 μmol photons m^−2^ s^−1^ light, 12/12 h light/dark photoperiod, ambient CO_2_, at 23 °C and 55% relative humidity. After 3 days of vernalization in distilled water at 4 °C, seeds were sown on soil and let to germinate for one week. After this period, uniform seedlings (20 per treatment) were individually transplanted into pots (4 plants per pot) containing soil and perlite. The growing substrate was composed as follows: neutral peat bog, green composted amendment, simple non-composted plant amendment, and expanded perlite (<5%), pH 7.5, electrical conductivity 0.30 dS/m, dry bulk density 167 kg/m^3^, and total porosity 90% *v*/*v*. To maintain optimal hydration, the plants were watered every other day (100 mL/pot) through an automated irrigation system. The seedlings were maintained under identical growing conditions and the experimental set-up described above was applied to all the experiments (experiment 1, experiment 2, and experiment 3, Figure S9), with each experiment being conducted only once.

### 5.2. Synthesis of Aminoselenide 4 [1-(Phenylselanyl)propan-2-amine]

Sodium borohydride (46 mg, 1.2 mmol) was added to a suspension of diphenyl diselenide **1** (125 mg, 0.4 mmol) in dry EtOH (3 mL) under an inert atmosphere (N_2_) at 0 °C. Then, 2-methylaziridine **3** (56 mg, 0.96 mmol) was slowly added and the mixture was allowed to warm to room temperature and then stirred for 2 h. Afterward, the reaction was diluted with EtOAc (10 mL) and saturated *aq.* NH_4_Cl (5 mL) was added. The organic phase was collected and the aqueous phase was extracted with EtOAc (2 × 10 mL). The combined organic phases were dried over Na_2_SO_4_ and concentrated under reduced pressure. The residue was purified by flash column chromatography (petroleum ether:EtOAc 1:2) to yield 1-(phenylselanyl)propan-2-amine **4** (89 mg, 51%). ^1^H NMR (400 MHz, CDCl_3_): *δ* (ppm) 1.19 (3H, dd, *J* = 6.3, 12.4 Hz), 2.45 (2H, bs, NH_2_), 2.84 (1H, dd, *J* = 7.7, 12.2 Hz, C**H_a_**H_b_Se), 3.05 (1H, dd, *J* = 4.8, 12.2 Hz, CH_a_**H_b_**Se), 3.08–3.16 (1H, m, C**H**NH_2_), 2.24–2.30 (3H, m), 7.52–7.56 (2H, m). ^13^C NMR (100 MHz, CDCl_3_): *δ* (ppm) 23.1, 38.7, 46.7, 127.0, 129.1, 130.0, 132.8. MS (ESI, positive): 216.5 [*M* + H]^+^.

### 5.3. Synthesis of Aminoselenide Hydrochloride (Se-AMA)

Se-AMA (**4** hydrochloride) was conveniently prepared upon the treatment of the parent aminoselenide **4** with an equimolar amount of hydrochloric acid (HCl 37%). A stock solution of Se-AMA hydrochloride was further prepared by adding ultrapure water.

### 5.4. Se-AMA Analysis

The 10 µM aqueous solution of Se-AMA was analyzed by HPLC-Q-ToF (Agilent 1260 Series equipped with an electrospray ionization (ESI) interface and coupled with Agilent 6530 Q-TOF mass spectrometer). The chromatographic separation was carried out on a column Poroshell120 CS-C18 (2.1 mm × 100 mm, 2.7 µm; Agilent, Santa Clara, CA, USA). The column was maintained at 30 °C and the injection volume was 1 μL. The mobile phases consisted of water (A) and methanol (B) and a liner gradient was applied passing from 50% A and 50% B to 5% A and 95% B in 15 min at a flow rate of 0.3 mL min^−1^. The composition was then returned to initial conditions and maintained for 15 min for equilibration. Mass spectrometry profiling was performed operating in positive ion mode with the following parameters: capillary voltage, 4000 V; fragmentor, 180 V; skimmer, 60 V; OCT 1 RF Vpp, 750 V; pressure of nebulizer, 20 psi; drying gas temperature, 325 °C; and sheath gas temperature, 400 °C. Nitrogen was used as sheath and drying gas at a flow rate of 10.0 and 12.0 L/min, respectively. Data were collected in the centroid mode and the mass range was set at *m*/*z* 50–1100 using the extended dynamic range. The accurate mass capability of the TOF analyzer was maintained by continuously spraying an external calibration solution (Agilent calibration solution A) to recalibrate the mass axis, ensuring mass accuracy and reproducibility throughout the chromatographic run. Agilent MassHunter Workstation Acquisition Software Version B.05.01 and Qualitative Analysis Software Version B.07.00 were utilized for system control, data acquisition, and data processing.

The Se-AMA solution showed the presence of the molecule (molecular weight 217.1247) and it was analyzed before and after each experiment and proved to be stable over the whole application period.

### 5.5. Se-AMA Treatments

The experiment was conducted according to a simple randomized design. Foliar-spraying treatments of Se-AMA were performed on 3-week-old plants using different doses and timings of administration (Figure S9). In experiment 1, three distinct groups of plants were treated through foliar spray with different concentrations of the tested molecule (25, 50, or 100 µM of Se-AMA dissolved in ultrapure water), whereas an additional set of plants were sprayed with a control solution (ultrapure water without Se-AMA) serving as controls. All the treatments were performed in the early morning, after sunrise, and were repeated every day until significant effects of Se-AMA on gas exchange variables were detected. In experiment 2, the plants were either treated with a control solution or with a higher Se-AMA concentration compared to that employed in experiment 1 (300 μM), applied once a day (in the morning) for three consecutive days until the first significant effects were observed. To further investigate the endurance of the physiological alterations induced by Se-AMA, experiment 3 was performed by applying control or 300 µM Se-AMA treatments in the morning and monitoring the course of photosynthesis-related parameters throughout the day. All the treatments were performed by spraying 2.5 mL of the prepared solutions (with or without Se-AMA) on each plant (10 mL/pot). To avoid overlapping treatments, the spray was applied by spacing the individual pots apart and isolating single plants within each pot using a plastic shield.

### 5.6. Evaluation of Plant Photosynthetic Performance

Net photosynthesis (A, μmol m^−2^ s^−1^), stomatal conductance (g_s_, mmol m^−2^ s^−1^), internal CO_2_ (C_i_, μmol mol^−1^), and transpiration (E, mmol m^−2^s^−1^) were analyzed using the CIRAS-3 infrared gas analyzer (PP Systems, Amesbur, MA, USA). Gas exchange measurements were performed with a flow of 300 µmol CO_2_ s^−1^ within a growth cabinet set to an air temperature of 25 °C and light intensity of 130 µmol quanta m^−2^ s^−1^. The incoming air was adjusted to a CO_2_ concentration of 400 ppm and a relative humidity of 70%. Punctual point measurements of leaf gas exchange were taken in the afternoon (at 14:00 h) at 8 h, 10 days, and 12 days after the Se-AMA treatments (experiment 1) or after 3 days of the molecule application (experiment 2), as reported in Figure S9. Diurnal variation in the gas exchange parameters was further recorded following a single application of 300 µM Se-AMA by performing gas exchange analyses twice, at 14:00 h and 18:00 h (experiment 3, Figure S9). All the repeated measurements were performed on two selected leaves per plant, and a minimum of 4 homogeneous biological replicates were chosen for each treatment. The apparent carboxylation efficiency (A/C_i_, mol m^−2^ s^−1^) and intrinsic water use efficiency (WUE_i_, μmol mmol^−1^) were calculated from the raw data of each experiment, normalizing net photosynthesis (A) to intracellular CO_2_ concentration (C_i_) or to stomatal conductance (g_s_).

The effect of high concentrations of Se-AMA (300 µM) on plant photosynthetic performance was also evaluated by measuring the maximum quantum efficiency of Photosystem II (F_v_/F_m_). Two selected leaves of each plant (two measurements per plant and 4 plants per treatment) were dark-adapted with leaf clips (PPEA/LC, Hansatech Instrument, King’s Lynn, UK) for 20 min. Then, the F_v_/F_m_ was measured using a portable chlorophyll fluorescence monitoring system (Handy PEA, Hansatech Instrument, King’s Lynn, England). Punctual measurements were performed 3 days-AT (experiment 2), whereas daily fluctuations were assessed by conducting the analysis twice throughout the day, at 14:00 h and 18:00 h (experiment 3) (Figure S9).

### 5.7. Plant Growth Parameters

After performing the gas exchange analyses in experiments 1 and 3, both the control and treated plants were harvested, and the growth-related parameters were measured. Directly after clipping, the samples were weighted and pictured for determining the biomass fresh weight (FW) and the whole plant leaf area using the free software ImageJ (https://imagej.net/), respectively. After drying in the oven at 60 °C, the biomass dry weight (DW) was measured, and several growth parameters were calculated as follows: water content (FW-DW), plant dry matter (DW/FW), FW:DW ratio (FW/DW), and plant mass/area measured on the whole aerial part (DW/Plant total leaf area).

### 5.8. Leaf Pigments Detection

To rule out the phytotoxic impact of Se-AMA, the health of plants treated with 300 μM Se-AMA for three days (experiment 2) was assessed by measuring leaf pigments (chlorophylls, flavonoids, and anthocyanins) and the nitrogen balance index (NBI) using a non-destructive measurement device (Dualex METOS^®^, Press Instruments, Weiz, Austria). For each treatment, 4 biological replicates were analyzed, with two leaves per plant. Measurements were taken on both sides of each leaf, and the sum of data from the upper and lower sides was calculated and used for statistical analysis. The content of total chlorophyll, total flavonoids, and total anthocyanins was assessed in DUALEX units and reported as indexes.

### 5.9. Statistical Analysis

The impact of the Se-AMA treatments on photosynthetic variables and the differences between time points were assessed using two-way repeated measures ANOVA, with time as the repeated factor (experiments 1 and 3, Figure 1 and Figure 5). Physiological data obtained in experiment 2 (Figure 3) were analyzed through an unpaired *t*-test with a 95% confidence level (*p* < 0.05). Plant growth data were analyzed using either an unpaired *t*-test or one-way ANOVA depending on whether a single dose or multiple doses of Se-AMA were applied. All the statistical analyses were supplemented by Tukey’s multiple comparison test with individual variances computed for each comparison.

## Data Availability

Data are contained within the article and Supplementary Materials.

## Supplementary Materials

The following supporting information can be downloaded at https://www.mdpi.com/article/10.3390/plants14020258/s1, Figure S1. Impact of Se-AMA on physiological performances of *Arabidopsis thaliana.* Detail of the gas exchange analysis performed on plants either treated with a mock solution (0 µM Se-AMA) or with different concentrations of Se-AMA (25, 50, or 100 µM) performed at 8 hours after treatment (AT), 10 days-AT and 12 days-AT: intracellular CO_2_ concentration (C_i_) (a) and intrinsic water use efficiency (WUE_i_) (b). Data are presented as means ± SD from four biological replicates, with different letters indicating significant differences between Se-AMA treatments at each time point (two-way ANOVA with repeated measurements, followed by a post-hoc Tukey test, *p* < 0.05, *n* = 4). A comprehensive summary of the statistical analysis is provided in Table S1, including details on the effect of time on photosynthetic performance for each treatment. Figure S2. Impact of different doses of Se-AMA on *Arabidopsis thaliana* biomass parameters. After 10 days of treatments, biomass related parameters relative to plants treated either with a mock solution (0 µM Se-AMA) or with different concentrations of Se-AMA (25, 50, or 100 µM) were assessed: plant water content (a), plant dry matter (b), plant area (c) and plant mass area (d). Data are presented as means ± SD from four biological replicates, with different letters indicating significant differences between Se-AMA treatments (One-way ANOVA, followed by a post-hoc Tukey test, *p* < 0.05, *n* = 4). Figure S3. Effect of high concentrations of Se-AMA on leaf pigments. After three days of treatment, total chlorophyll index (a), total flavonoid index (b) and total anthocyanin index were measured in leaves of control (0 µM Se-AMA) and treated plants (300 µM Se-AMA). The nitrogen balance index (NBI) was also evaluated. Data are presented as means ± SD, with stars indicating significant differences between control and Se-AMA treatment (unpaired *t*-test, * *p* < 0.05, ** *p* < 0.01, *** *p* < 0.001, *n* = 5). Figure S4. Impact of high doses of Se-AMA on physiological performances of *Arabidopsis thaliana.* Detail of the gas exchange analysis on plants either treated with a control solution (0 µM Se-AMA) or with 300 µM Se-AMA performed at 3 days after treatment (AT): intracellular CO_2_ concentration (C_i_) (a) and intrinsic water use efficiency (WUE_i_) (b). Data are presented as means ± SD, with stars indicating significant differences between control and Se-AMA treatment (unpaired *t*-test, * *p* < 0.05, ** *p* < 0.01, *** *p* < 0.001, *n* = 5). Figure S5. Impact of high doses of Se-AMA on the efficiency of PSII. After three days of treatment, the maximum quantum efficiency of Photosystem II (F_v_/F_m_) was assessed in plants either treated with a mock solution (0 µM Se-AMA) or with 300 µM Se-AMA. Data are presented as means ± SD, with stars indicating significant differences between control and Se-AMA treatment (unpaired *t*-test, * *p* < 0.05, ** *p* < 0.01, *** *p* < 0.001, *n* = 5). Figure S6. Impact of high doses of Se-AMA on *Arabidopsis thaliana* growth. After 10 days of treatments, growth related parameters relative to plants treated either with a mock solution (0 µM Se-AMA) or with different concentrations of Se-AMA (25, 50, or 100 µM) were assessed: plant water content (a), plant dry matter (b), plant area (c) and plant mass area (d). Data are presented as means ± SD from five biological replicates, with different letters indicating significant differences between Se-AMA treatments (One-way ANOVA, followed by a post-hoc Tukey test, *p* < 0.05, *n* = 5). Figure S7. Daily effect of Se-AMA on physiological performances of *Arabidopsis thaliana.* Details of the gas exchange analysis performed at 14:00 hours (h) and 18:00 h on plants subjected to a single treatment with a control solution or with Se-AMA at 300 µM: intracellular CO_2_ concentration (C_i_) (a) and intrinsic water use efficiency (WUE_i_) (b). Data are presented as means ± SD, with different letters indicating significant differences between Se-AMA treatments at each time point (two-way ANOVA with repeated measurements, followed by a post-hoc Tukey test, *p* < 0.05, *n* = 5). A comprehensive summary of the statistical analysis is provided in Table S2, including details on the effect of time on photosynthetic performance for each treatment. Figure S8. Daily effect of Se-AMA on the efficiency of PSII. Analysis of the maximum quantum efficiency of Photosystem II (Fv/Fm) performed at 14:00 hours (h) and 18:00 h on plants subjected to a single treatment with a control solution or with Se-AMA at 300 µM. Data are presented as means ± SD, with different letters indicating significant differences between Se-AMA treatments at each time point (two-way ANOVA with repeated measurements, followed by a post-hoc Tukey test, *p* < 0.05, *n* = 5). A comprehensive summary of the statistical analysis is provided in Table S2, including details on the effect of time on PSII efficiency for each treatment. Figure S9. Schematic representation of the experiments conducted to study the effect of Se-AMA application on the photosynthetic performance of *Arabidopsis thaliana* plants. In experiment 1, plants either treated with a control solution (0 µM Se-AMA) or with different concentrations of the molecule (25, 50 and 100 µM Se-AMA), were subjected to gas exchange analysis at 8 hours after treatment (AT), 10 days-AT and 12 days-AT (a). In experiment 2, gas exchange analysis, F_v_/F_m_ measurements and leaf pigments detection were performed after 3 days of treatment with 300 M Se-AMA (b). In experiment 3, the effect of a single application of Se-AMA on daily physiological performances was assessed by performing gas exchange analysis twice throughout the day, at 14:00 hours (h) and 18:00 h (c). Table S1. Results of two-way RM-ANOVA showing the effect of Se-AMA treatments on different gas exchange parameters at three time points: 8 hours-AT, 10 days-AT and 12 days-AT. Values are means ± SD and different letters indicate significant differences among different time points within each treatment (Tukey pairwise comparison, *p* < 0.05). Abbreviations: net photosynthesis (A), stomatal conductance (g_s_), apparent carboxylation efficiency (A/C_i_), transpiration rate (E), intercellular CO_2_ concentration (C_i_); intrinsic water use efficiency (WUE_i_) and maximum quantum efficiency of Photosystem II (F_v_/F_m_). Table S2. Results of two-way RM-ANOVA showing the daily effect of Se-AMA on different gas exchanges parameters measured at two time points: 14:00 h and 18:00 h. Values are means ± SD and different letters indicate significant differences among different time points within each treatment (Tukey pairwise comparison, *p* < 0.05). Abbreviations: net photosynthesis (A), stomatal conductance (g_s_), apparent carboxylation efficiency (A/C_i_), transpiration rate (E), intercellular CO_2_ concentration (C_i_); intrinsic water use efficiency (WUE_i_) and maximum quantum efficiency of Photosystem II (F_v_/F_m_).

## References

[1] Wu A., Hammer G.L., Doherty A., von Caemmerer S., Farquhar G.D. (2019). Quantifying Impacts of Enhancing Photosynthesis on Crop Yield. Nat. Plants.

[2] Iñiguez C., Aguiló-Nicolau P., Galmés J. (2021). Improving Photosynthesis through the Enhancement of Rubisco Carboxylation Capacity. Biochem. Soc. Trans..

[3] DiMario R.J., Clayton H., Mukherjee A., Ludwig M., Moroney J.V. (2017). Plant Carbonic Anhydrases: Structures, Locations, Evolution, and Physiological Roles. Mol. Plant.

[4] Meldrum N.U., Roughton F.J.W. (1933). The state of carbon dioxide in blood. J. Physiol..

[5] Hewett-Emmett D., Tashian R.E. (1996). Functional Diversity, Conservation, and Convergence in the Evolution of the α-, β-, and γ-Carbonic Anhydrase Gene Families. Mol. Phylogenet. Evol..

[6] Rudenko N.N., Ivanov B.N. (2021). Unsolved Problems of Carbonic Anhydrases Functioning in Photosynthetic Cells of Higher C3 Plants. Biochemistry.

[7] Tholen D., Zhu X.G. (2011). The Mechanistic Basis of Internal Conductance: A Theoretical Analysis of Mesophyll Cell Photosynthesis and CO_2_ Diffusion. Plant Physiol..

[8] Ogée J., Wingate L., Genty B. (2018). Estimating Mesophyll Conductance from Measurements of C^18^OO Photosynthetic Discrimination and Carbonic Anhydrase Activity. Plant Physiol..

[9] Momayyezi M., McKown A.D., Bell S.C.S., Guy R.D. (2020). Emerging Roles for Carbonic Anhydrase in Mesophyll Conductance and Photosynthesis. Plant J..

[10] Wu Y., Rao S. (2023). Root-Derived Bicarbonate Assimilation in Plants.

[11] Perez-Martin A., Michelazzo C., Torres-Ruiz J.M., Flexas J., Fernández J.E., Sebastiani L., Diaz-Espejo A. (2014). Regulation of Photosynthesis and Stomatal and Mesophyll Conductance under Water Stress and Recovery in Olive Trees: Correlation with Gene Expression of Carbonic Anhydrase and Aquaporins. J. Exp. Bot..

[12] Wang Y., Wang Y., Tang Y., Zhu X.G. (2022). Stomata Conductance as a Goalkeeper for Increased Photosynthetic Efficiency. Curr. Opin. Plant Biol..

[13] Zhou L., Xiang X., Ji D., Chen Q., Ma T., Wang J., Liu C. (2024). A Carbonic Anhydrase, ZmCA4, Contributes to Photosynthetic Efficiency and Modulates CO_2_ Signaling Gene Expression by Interacting with Aquaporin ZmPIP2;6 in Maize. Plant Cell Physiol..

[14] Rudenko N.N., Ignatova L.K., Zhurikova E.M., Yanyushin M.F., Ivanov B.N. (2018). The multiplicity of functions of carbonic anhydrases in higher plants. Carbonic Anhydrases: Biochemistry, Mechanism of Action and Therapeutic Applications.

[15] Ortega M.A., De Leon-Oliva D., Gimeno-Longas M.J., Boaru D.L., Fraile-Martinez O., García-Montero C., de Castro A.V., Barrena-Blázquez S., López-González L., Amor S. (2024). Vascular Calcification: Molecular Networking, Pathological Implications and Translational Opportunities. Biomolecules.

[16] Akocak S., Supuran C.T. (2019). Activation of α-, β-, γ-δ-, ζ- and η-Class of Carbonic Anhydrases with Amines and Amino Acids: A Review. J. Enzym. Inhib. Med. Chem..

[17] Mishra C.B., Tiwari M., Supuran C.T. (2020). Progress in the Development of Human Carbonic Anhydrase Inhibitors and Their Pharmacological Applications: Where Are We Today?. Med. Res. Rev..

[18] Barresi E., Ravichandran R., Germelli L., Angeli A., Baglini E., Salerno S., Marini A.M., Costa B., Da Pozzo E., Martini C. (2021). Carbonic Anhydrase Activation Profile of Indole-Based Derivatives. J. Enzym. Inhib. Med. Chem..

[19] Tanini D., Carradori S., Capperucci A., Lupori L., Zara S., Ferraroni M., Ghelardini C., Di Cesare Mannelli L., Micheli L., Lucarini E. (2021). Chalcogenides-incorporating carbonic anhydrase inhibitors concomitantly revertedoxaliplatin-induced neuropathy and enhanced antiproliferative action. Eur. J. Med. Chem..

[20] Nocentini A., Angeli A., Carta F., Winum J.Y., Zalubovskis R., Carradori S., Capasso C., Donald W.A., Supuran C.T. (2021). Reconsidering Anion Inhibitors in the General Context of Drug Design Studies of Modulators of Activity of the Classical Enzyme Carbonic Anhydrase. J. Enzym. Inhib. Med. Chem..

[21] Zhou X.-q., Ma X.-l., Ariffin N.S. (2024). The Potential of Carbonic Anhydrase Enzymes as a Novel Target for Anti-Cancer Treatment. Eur. J. Pharmacol..

[22] Temperini C., Scozzafava A., Vullo D., Supuran C.T. (2006). Carbonic Anhydrase Activators. Activation of Isozymes I, II, IV, VA, VII, and XIV with L- and D-Histidine and Crystallographic Analysis of Their Adducts with Isoform II: Engineering Proton-Transfer Processes within the Active Site of an Enzyme. Chem.-A Eur. J..

[23] Tanini D., Capperucci A., Supuran C.T., Angeli A. (2019). Sulfur, Selenium and Tellurium Containing Amines Act as Effective Carbonic Anhydrase Activators. Bioorg. Chem..

[24] Capperucci A., Coronnello M., Salvini F., Tanini D., Dei S., Teodori E., Giovannelli L. (2021). Synthesis of Functionalised Organochalcogenides and in Vitro Evaluation of Their Antioxidant Activity. Bioorg. Chem..

[25] Kromdijk J., Long S.P. (2016). One Crop Breeding Cycle from Starvation? How Engineering Crop Photosynthesis for Rising CO_2_ and Temperature Could Be One Important Route to Alleviation. Proc. R. Soc. B Biol. Sci..

[26] Malhi G.S., Kaur M., Kaushik P. (2021). Impact of Climate Change on Agriculture and Its Mitigation Strategies: A Review. Sustainability.

[27] Simkin A.J., McAusland L., Lawson T., Raines C.A. (2017). Overexpression of the RieskeFeS protein increases electron transport rates and biomass yield. Plant Physiol..

[28] Cardoso A.A., Gori A., Da-Silva C.J., Brunetti C. (2020). Abscisic acid biosynthesis and signaling in plants: Key targets to improve water use efficiency and drought tolerance. Appl. Sci..

[29] Provensi G., Costa A., Rani B., Becagli M.V., Vaiano F., Passani M.B., Tanini D., Capperucci A., Carradori S., Petzer J.P. (2022). New β-arylchalcogeno amines with procognitive properties targeting Carbonic Anhydrases and Monoamine Oxidases. Eur. J. Med. Chem..

[30] Tanini D., Borgogni C., Capperucci A. (2019). Mild and selective silicon-mediated access to enantioenriched 1,2-mercaptoamines and β-amino arylchalcogenides. New J. Chem..

[31] Dewar R., Mauranen A., Mäkelä A., Hölttä T., Medlyn B., Vesala T. (2018). New Insights into the Covariation of Stomatal, Mesophyll and Hydraulic Conductances from Optimization Models Incorporating Nonstomatal Limitations to Photosynthesis. New Phytol..

[32] Sakoda K., Yamori W., Groszmann M., Evans J.R. (2021). Stomatal, Mesophyll Conductance, and Biochemical Limitations to Photosynthesis during Induction. Plant Physiol..

[33] Petrík P., Petek-petrik A., Mukarram M., Schuldt B., Lamarque L.J. (2023). Emerging Voices in Botany Leaf Physiological and Morphological Constraints of Water- Use Efficiency in C3 Plants. AoB Plants.

[34] Yamori W. (2019). Photosynthesis and Respiration.

[35] Simonin K.A., Burns E., Choat B., Barbour M.M., Dawson T.E., Franks P.J. (2015). Increasing leaf hydraulic conductance with transpiration rate minimizes the water potential drawdown from stem to leaf. J. Exp. Bot..

[36] Thompson A.J., Andrews J., Mulholland B.J., McKee J.M., Hilton H.W., Horridge J.S., Farquhar G.D., Smeeton R.C., Smillie I.R., Black C.R. (2007). Overproduction of abscisic acid in tomato increases transpiration efficiency and root hydraulic conductivity and influences leaf expansion. Plant Physiol..

[37] Wang C., Hu H., Qin X., Zeise B., Xu D., Rappel W.J., Boron W.F., Schroeder J.I. (2015). Reconstitution of CO_2_ Regulation of SLAC1 Anion Channel and Function of CO_2_-Permeable PIP2;1 Aquaporin as CARBONIC ANHYDRASE4 Interactor. Plant Cell.

[38] Del Carmen Martinez-Ballesta M., Carvajal M. (2014). New Challenges in Plant Aquaporin Biotechnology. Plant Sci..

[39] Sadok W., Lopez J.R., Smith K.P. (2021). Transpiration Increases under High-Temperature Stress: Potential Mechanisms, Trade-Offs and Prospects for Crop Resilience in a Warming World. Plant Cell Environ..

