# Supplementary material for: Exploring the Potential of Selenium-Containing Amine (Se-AMA) to Enhance Photosynthesis and Leaf Water Content: New Avenues for Carbonic Anhydrase Modulation in Arabidopsis thaliana
Source: Plants (Basel). 2025 Jan 17;14(2):258. doi: 10.3390/plants14020258 (PMC11768400; doi:10.3390/plants14020258)
Supplement: Supplementary file 1 [file plants-14-00258-s001.zip › plants-3419106-supplementary.pdf]

Supplementary Material

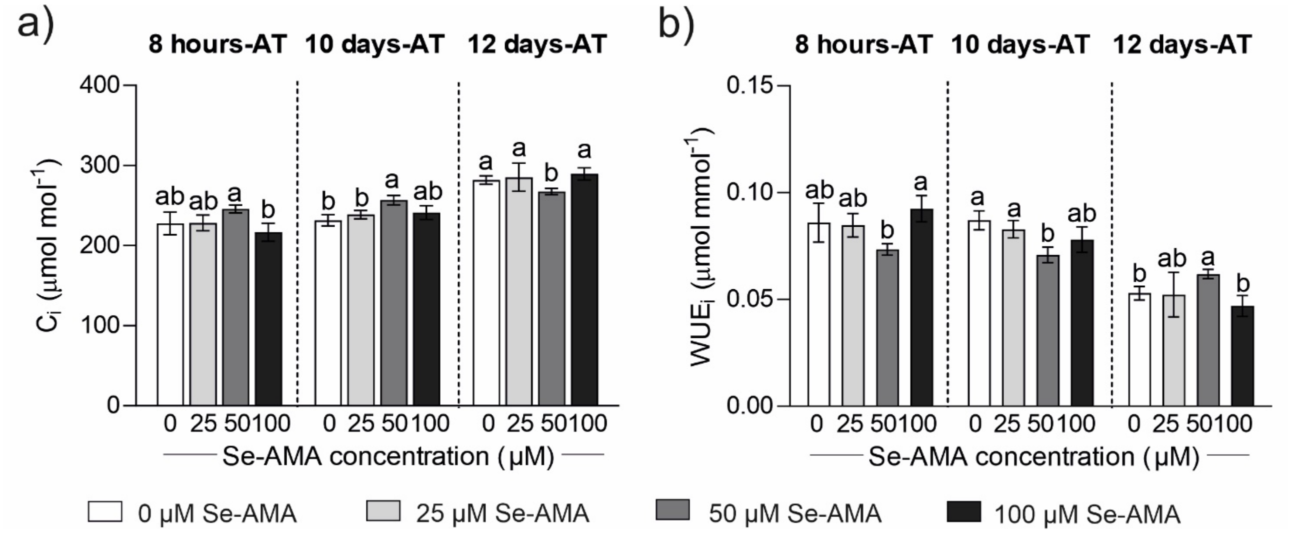

Figure S1. Impact of Se-AMA on physiological performances of *Arabidopsis thaliana.* Detail of the gas exchange analysis performed on plants either treated with a mock solution (0 µM Se-AMA) or with different concentrations of Se-AMA (25, 50, or 100 µM) performed at 8 hours after treatment (AT), 10 days-AT and 12 days-AT: intracellular CO_2 concentration (C_i) (a) and intrinsic water use efficiency (WUE_i) (b). Data are presented as means ± SD from four biological replicates, with different letters indicating significant differences between Se-AMA treatments at each time point (two-way ANOVA with repeated measurements, followed by a post-hoc Tukey test, p < 0.05, n=4). A comprehensive summary of the statistical analysis is provided in Table S1, including details on the effect of time on photosynthetic performance for each treatment.

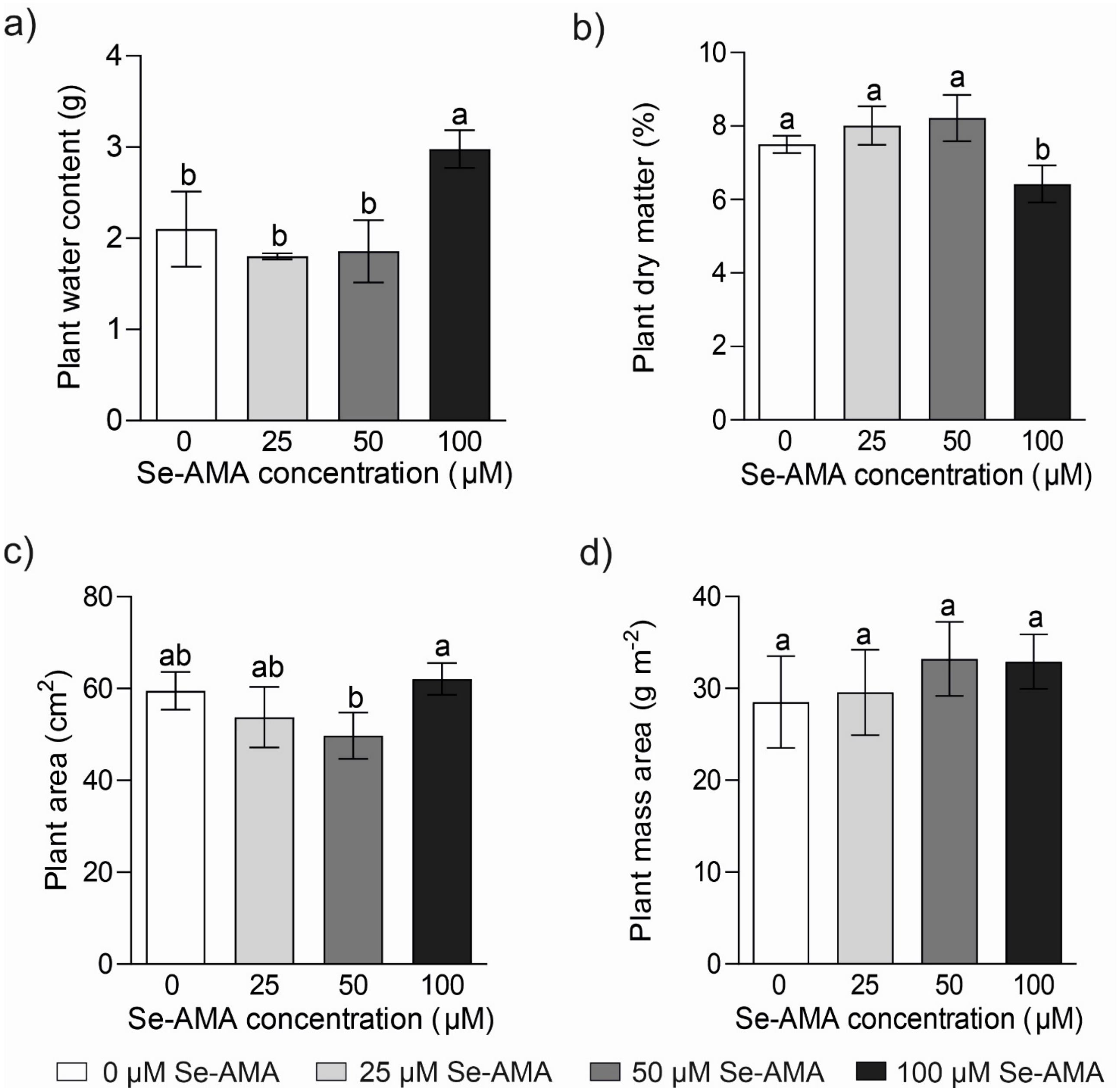

Figure S2. Impact of different doses of Se-AMA on *Arabidopsis thaliana* biomass parameters. After 10 days of treatments, biomass related parameters relative to plants treated either with a mock solution (0 μM Se-AMA) or with different concentrations of Se-AMA (25, 50, or 100 μM) were assessed: plant water content (a), plant dry matter (b), plant area (c) and plant mass area (d). Data are presented as means ± SD from four biological replicates, with different letters indicating significant differences between Se-AMA treatments (One-way ANOVA, followed by a post-hoc Tukey test, $p < 0.05$).

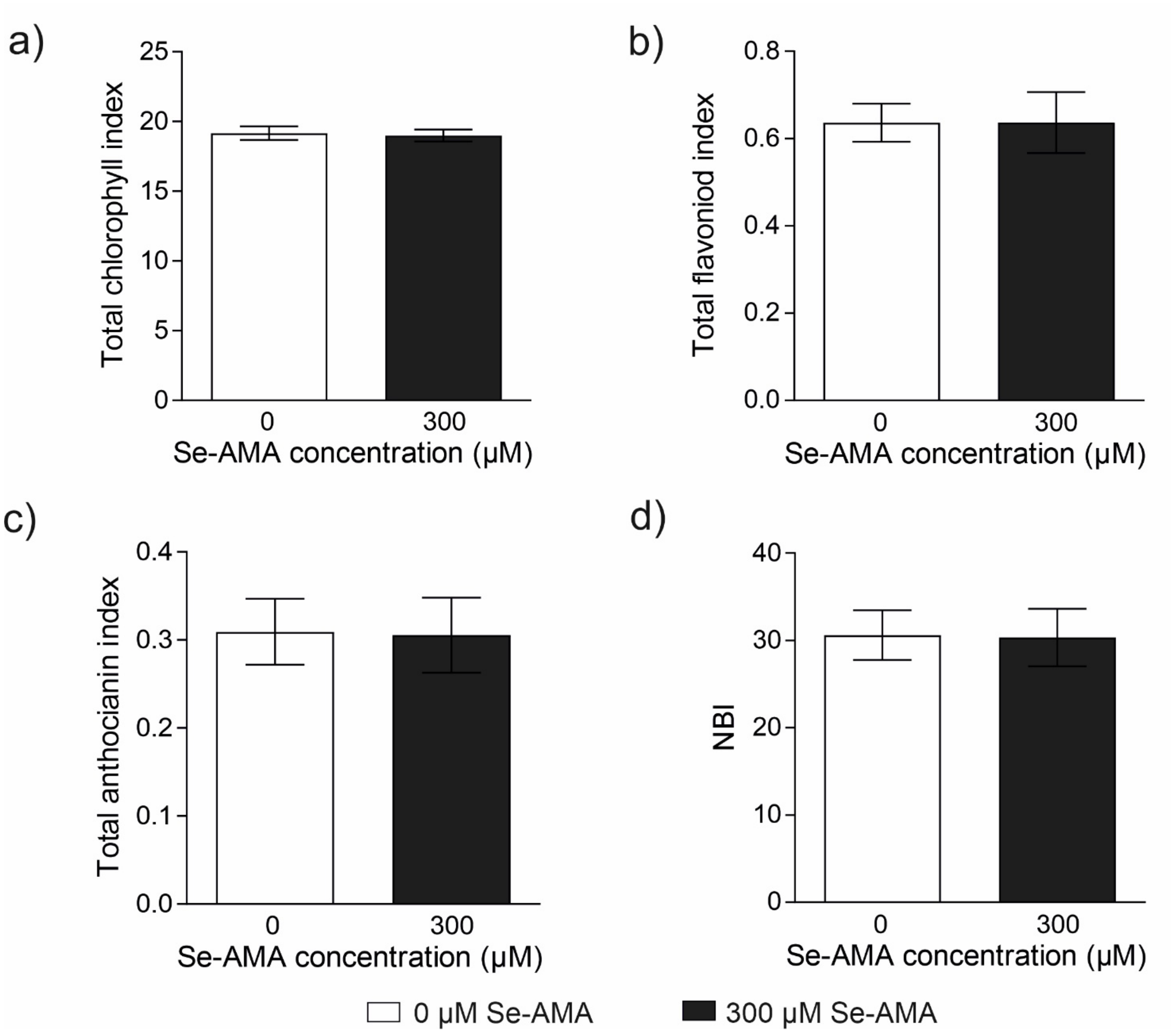

Figure S3. Effect of high concentrations of Se-AMA on leaf pigments. After three days of treatment, total chlorophyll index (a), total flavonoid index (b) and total anthocyanin index were measured in leaves of control (0 µM Se-AMA) and treated plants (300 µM Se-AMA). The nitrogen balance index (NBI) was also evaluated. Data are presented as means ± SD, with stars indicating significant differences between control and Se-AMA treatments (unpaired t-test, * $p < 0.05$, ** $p < 0.01$, *** $p < 0.001$, n=5).

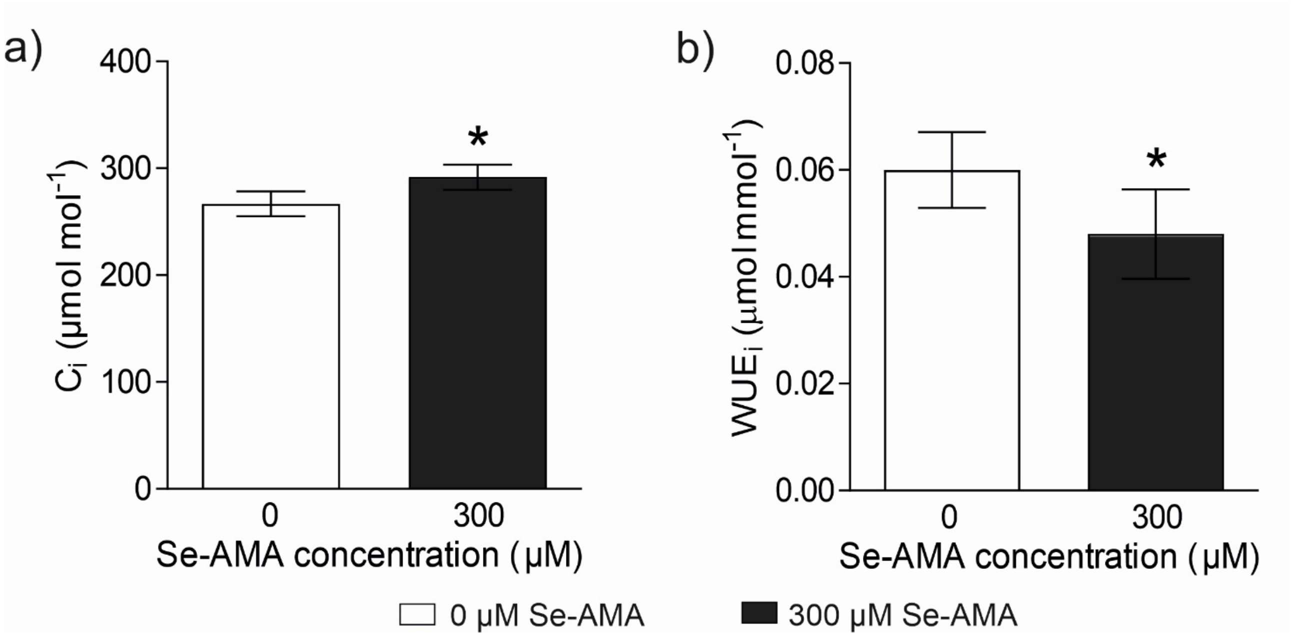

Figure S4. Impact of high doses of Se-AMA on physiological performances of *Arabidopsis thaliana*. Detail of the gas exchange analysis on plants either treated with a control solution (0 µM Se-AMA) or with 300 µM Se-AMA performed at 3 days after treatment (AT): intracellular CO_2 concentration (C_i) (a) and intrinsic water use efficiency (WUE_i) (b). Data are presented as means ± SD, with stars indicating significant differences between control and Se-AMA treatments (unpaired t-test, * $p < 0.05$, ** $p < 0.01$, *** $p < 0.001$, n=5).

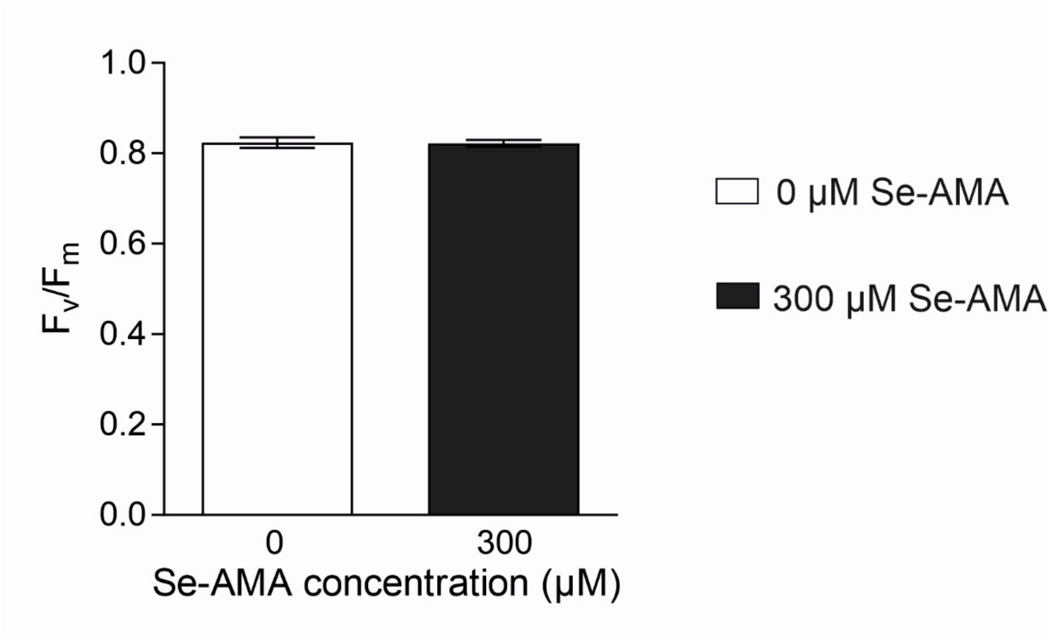

Figure S5. Impact of high doses of Se-AMA on the efficiency of PSII. After three days of treatment, the maximum quantum efficiency of Photosystem II (F_v/F_m) was assessed in plants either treated with a mock solution (0 µM Se-AMA) or with 300 µM Se-AMA. Data are presented as means ± SD, with stars indicating significant differences between control and Se-AMA treatments (unpaired t-test, * $p < 0.05$, ** $p < 0.01$, *** $p < 0.001$, n=5).

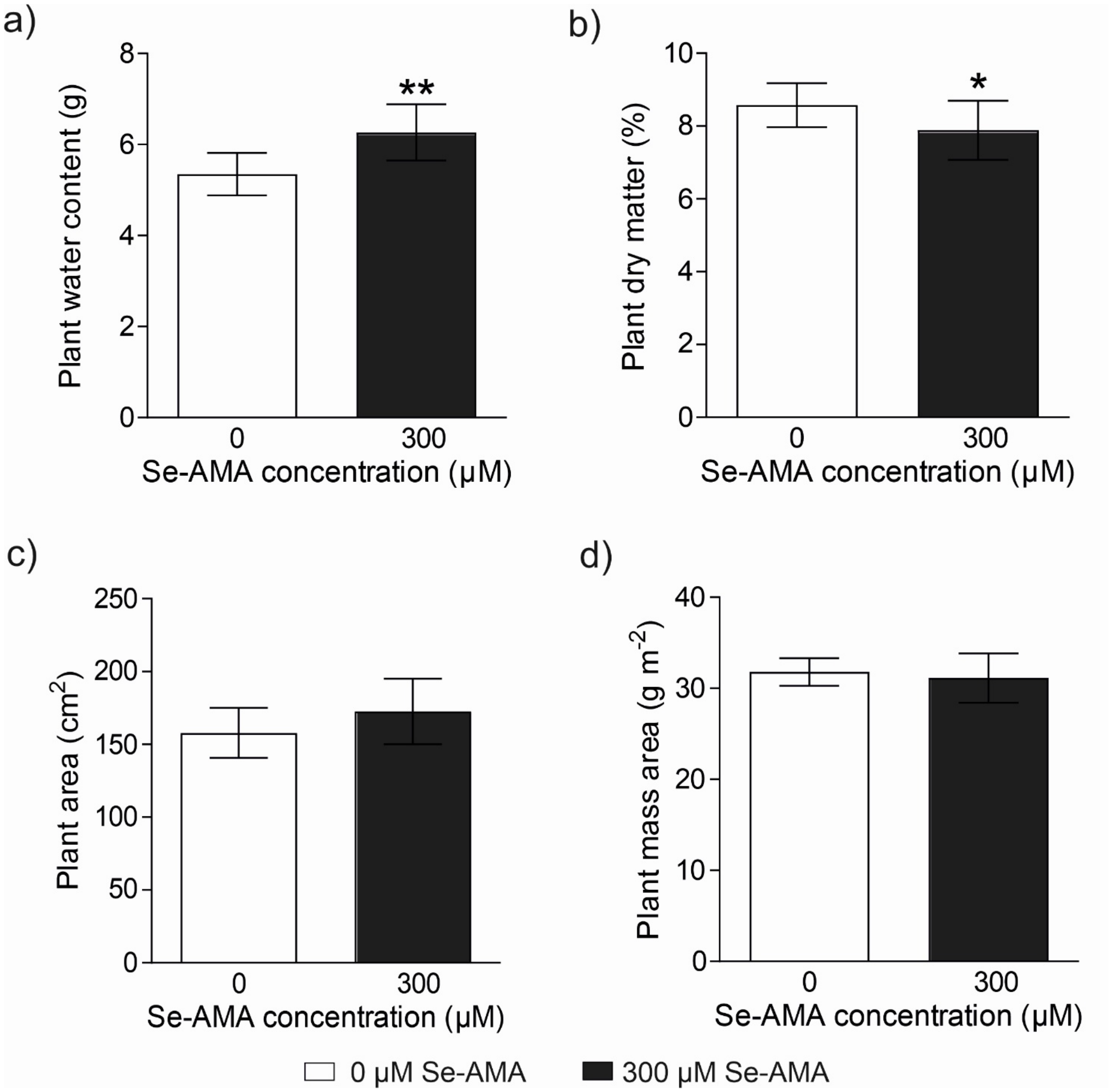

Figure S6. Impact of high doses of Se-AMA on *Arabidopsis thaliana* growth. After 10 days of treatments, growth related parameters relative to plants treated either with a mock solution (0 µM Se-AMA) or with different concentrations of Se-AMA (25, 50, or 100 µM) were assessed: plant water content (a), plant dry matter (b), plant area (c) and plant mass area (d). Data are presented as means ± SD from four biological replicates, with different letters indicating significant differences between Se-AMA treatments (One-way ANOVA, followed by a post-hoc Tukey test, $p < 0.05$, n=5).

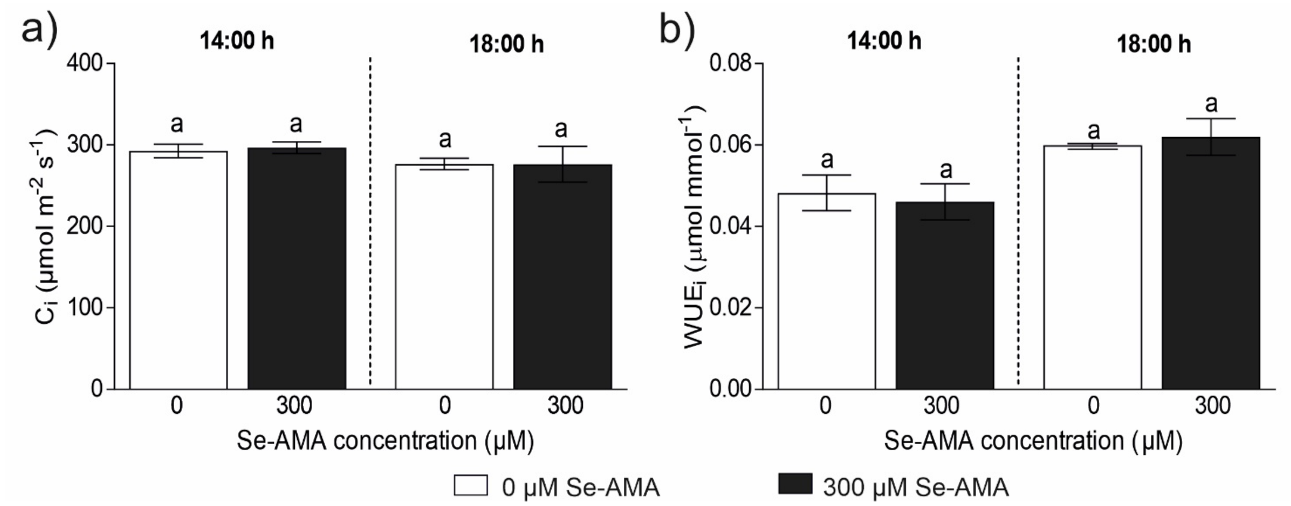

Figure S7. Daily effect of Se-AMA on physiological performances of *Arabidopsis thaliana.* Details of the gas exchange analysis performed at 14:00 hours (h) and 18:00 h on plants subjected to a single treatment with a control solution or with Se-AMA at 300 µM: intracellular CO_2 concentration (C_i) (a) and intrinsic water use efficiency (WUE_i) (b). Data are presented as means ± SD, with different letters indicating significant differences between Se-AMA treatments at each time point (two-way ANOVA with repeated measurements, followed by a post-hoc Tukey test, $p < 0.05$, n=5). A comprehensive summary of the statistical analysis is provided in Table S2, including details on the effect of time on photosynthetic performance for each treatment.

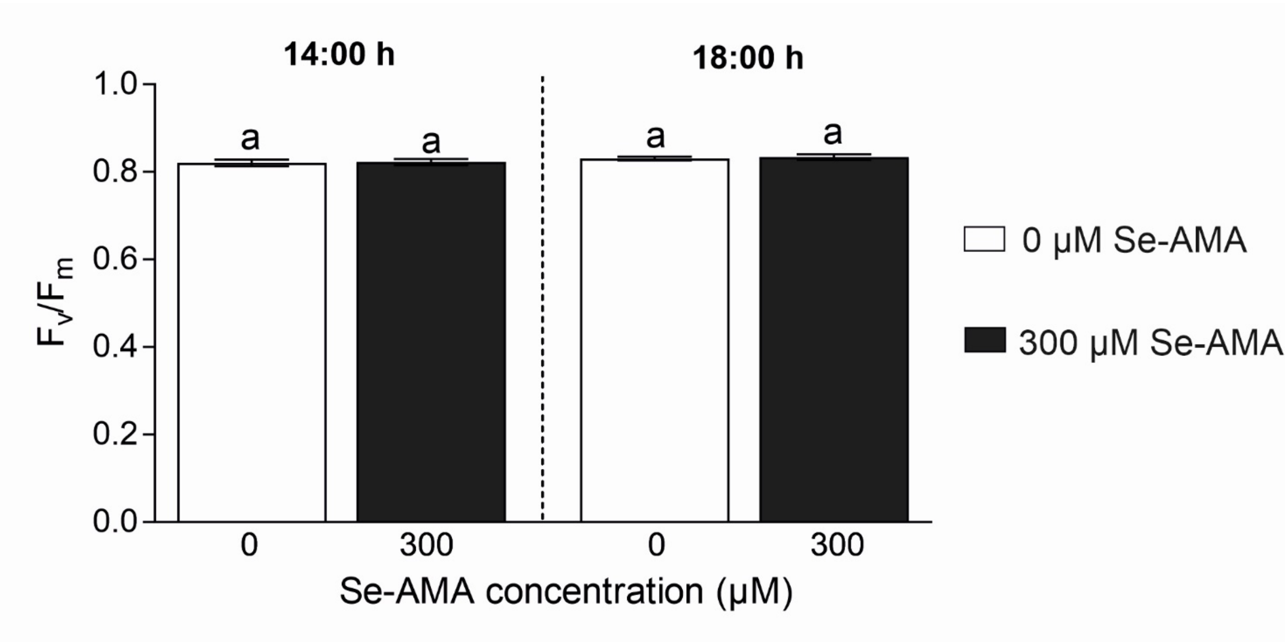

Figure S8. Daily effect of Se-AMA on the efficiency of PSII. Analysis of the maximum quantum efficiency of Photosystem II (Fv/Fm) performed at 14:00 hours (h) and 18:00 h on plants subjected to a single treatment with a control solution or with Se-AMA at 300 µM. Data are presented as means ± SD, with different letters indicating significant differences between Se-AMA treatments at each time point (two-way ANOVA with repeated measurements, followed by a post-hoc Tukey test, $p < 0.05$, n=5). A comprehensive summary of the statistical analysis is provided in Table S2, including details on the effect of time on PSII efficiency for each treatment.

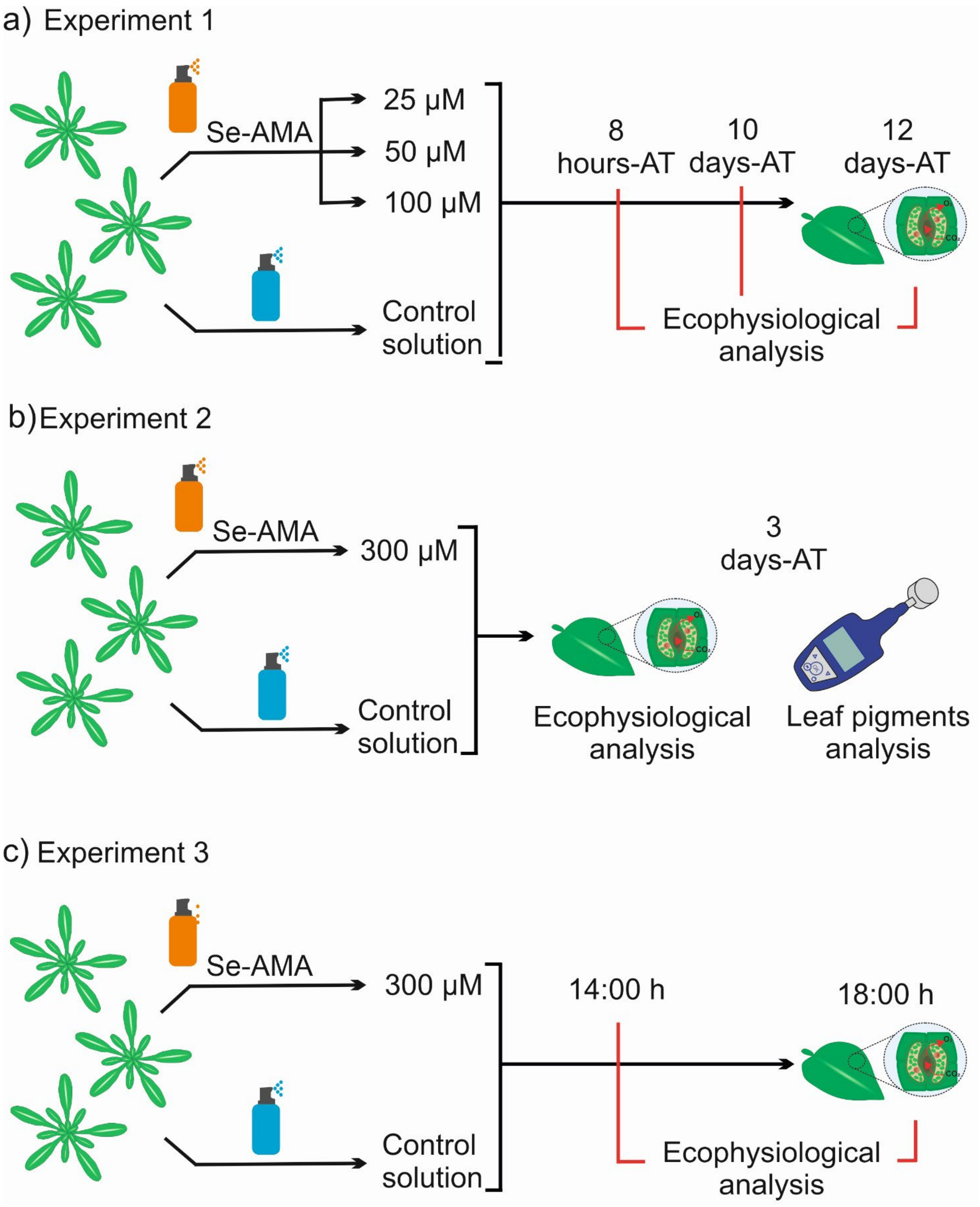

Figure S9. Schematic representation of the experiments conducted to study the effect of Se-AMA application on the photosynthetic performance of *Arabidopsis thaliana* plants. In experiment 1, plants either treated with a control solution (0 µM Se-AMA) or with different concentrations of the molecule (25, 50 and 100 µM Se-AMA), were subjected to gas exchange analysis at 8 hours after treatment (AT), 10 days-AT and 12 days-AT (a). In experiment 2, gas exchange analysis, F_v/F_m measurements and leaf pigments detection were performed after 3 days of treatment with 300 M Se-AMA (b). In experiment 3, the effect of a single application of Se-AMA on daily physiological performances was assessed by performing gas exchange analysis twice throughout the day, at 14:00 hours (h) and 18:00 h (c).

Treatment	Time	A	g_s	A/C_i	E	C_i	WUE_i
0 µM Se-AMA	8 hours-AT	15.400 ± 0.712 A	181.333 ± 21.312 B	0.068 ± 0.005 A	4.643 ± 0.492 B	227,667 ± 14.197 AB	0,086 ± 0.009 A
	10 days-AT	12.528 ± 0.551 B	147.778 ± 11.597 B	0.054 ± 0.002 B	3.348 ± 0.139 C	231,444 ± 6.905 B	0,087 ± 0.004 A
	12 days-AT	13.563 ± 0.144 B	257.875 ± 16.525 A	0.048 ± 0.001 C	5.965 ± 0.238 A	281,875 ± 5.218 A	0,053 ± 0.003 B
25 µM Se-AMA	8 hours-AT	15.867 ± 0.967 A	187.667 ± 9.568 A	0.070 ± 0.007 A	4.823 ± 0.147 A	228,333 ± 9.741 AB	0,085 ± 0.005 A
	10 days-AT	12.417 ± 1.017 B	160.167 ± 16.903 B	0.052 ± 0.003 B	3.505 ± 0.328 B	238,667 ± 5.039 B	0,083 ± 0.004 A
	12 days-AT	11.283 ± 0.966 B	230.500 ± 38.758 A	0.040 ± 0.005 C	5.462 ± 0.720 A	285,333 ± 17.485 A	0,052 ± 0.010 B
50 µM Se-AMA	8 hours-AT	15.433 ± 0.544 A	210.333 ± 2.625 A	0.063 ± 0.003 A	5.497 ± 0.070 A	245,667 ± 4.922 A	0,073 ± 0.003 A
	10 days-AT	13.133 ± 0.419 B	186.500 ± 13.096 A	0.051 ± 0.002 B	3.778 ± 0.146 B	256,667 ± 5.793 A	0,071 ± 0.004 B
	12 days-AT	13.200 ± 0.374 B	223.167 ± 17.513 A	0.049 ± 0.002 B	5.412 ± 0.130 A	267,500 ± 4.143 B	0,062 ± 0.002 C
100 µM Se-AMA	8 hours-AT	16.533 ± 0.531 A	179.333 ± 6.848 B	0.077 ± 0.006 A	4.453 ± 0.299 B	216,667 ± 11.116 B	0,092 ± 0.006 A
	10 days-AT	16.500 ± 0.402 A	219.000 ± 22.450 A	0.068 ± 0.001 A	4.317 ± 0.343 B	241,167 ± 8.778 AB	0,078 ± 0.006 A
	12 days-AT	15.433 ± 0.403 A	334.333 ± 40.592 A	0.053 ± 0.001 B	6.913 ± 0.434 A	289,500 ± 7.627 A	0,047 ± 0.005 B
Significance							
Treatment		<0.0001	<0.0001	<0.0001	<0.0001	0.0007	0.1338
Time		<0.0001	<0.0001	<0.0001	<0.0001	<0.0001	<0.0001
Treat x Time		<0.0001	<0.0001	<0.0001	<0.0001	<0.0001	<0.0001

Table S1. Results of two-way RM-ANOVA showing the effect of Se-AMA treatments on different gas exchange parameters at three time points: 8 hours-AT, 10 days-AT and 12 days-AT. Values are means ± SD and different letters indicate significant differences among different time points within each treatment (Tukey pairwise comparison, $p<0.05$). Abbreviations: net photosynthesis (A), stomatal conductance (g_s), apparent carboxylation efficiency (A/C_i), transpiration rate (E), intercellular CO_2 concentration (C_i); intrinsic water use efficiency (WUE_i) and maximum quantum efficiency of Photosystem II (F_v/F_m).

Treatment	Time	A	g_s	A/C_i	E	C_i	WUE_i	F_v/F_m
0 µM Se-AMA	14:00 h	9,457 ± 0.062 A	196.700 ± 16.202 A	0.032 ± 0.003 A	4.771 ± 0.254 A	292.800 ± 8.389 A	0,048 ± 0.004 B	0.820 ± 0.007 B
	18:00 h	11,038 ± 0.406 A	143.400 ± 7.495 B	0.032 ± 0.002 A	4.018 ± 0.337 B	276.800 ± 7.005 B	0,060 ± 0.002 A	0.830 ± 0.004 A
300 µM Se-AMA	14:00 h	8.967 ± 0.537 A	246.417 ± 24.010 A	0.037 ± 0.002 A	5.355 ± 0.439 A	296.792 ± 7.183 A	0.045 ± 0.004 B	0.823 ± 0.007 B
	18:00 h	10.175 ± 0.422 B	171.700 ± 20.117 B	0.037 ± 0.003 A	4.081 ± 0.344 B	276.500 ± 22.031 B	0,062 ± 0.008 A	0.834 ± 0.007 A
Significance								
Treatment		0.0012	0.0064	0.0081	0.0992	0.7986	0.9677	0.4599
Time		0.0033	<0.0001	0.9242	<0.0001	0.0022	<0.0001	0.0002
Treat x Time		0.2878	0.0301	0.8784	0.0921	0.6135	0.1425	0.8316

Table S2. Results of two-way RM-ANOVA showing the daily effect of Se-AMA on different gas exchanges parameters measured at two time points: 14:00 h and 18:00 h. Values are means ± SD and different letters indicate significant differences among different time points within each treatment (Tukey pairwise

comparison, $p<0.05$). Abbreviations: net photosynthesis (A), stomatal conductance (g_s), apparent carboxylation efficiency (A/C_i), transpiration rate (E), intercellular CO_2 concentration (C_i); intrinsic water use efficiency (WUE_i) and maximum quantum efficiency of Photosystem II (F_v/F_m).
